# Measurement of the Vaginal Pressure Profile with the Femfit® and Leakage Events Using a Newly Developed Pad Test during Selected Sports Activities: A Pilot Study

**DOI:** 10.1007/s00192-025-06051-y

**Published:** 2025-01-21

**Authors:** Magdaléna Hagovská, Alena Bukova, Jan Svihra

**Affiliations:** 1 Department of Physiatry, Balneology and Medical Rehabilitation, Institution - Faculty of Medicine, PJ Safarik University, Kosice, Slovak Republic; 2 Institute of Physical Education and Sport, Institution - PJ Safarik University, Kosice, Slovak Republic; 3 Department of Urology, Institution - Jessenius Faculty of Medicine, Comenius University Bratislava, Martin, Slovak Republic

**Keywords:** Vaginal pressure profile, Newly developed innovative pad weighing test, Female athletes, Urine leakage

## Abstract

**Introduction and Hypothesis:**

Measuring changes in the vaginal pressure profile (VPP) with the Femfit® by JUNOFEM during various sports will help improve understanding of the magnitude of pressures generated during such activities and the pelvic floor’s response to those pressures. This could aid in selecting safe exercises for women with pelvic floor dysfunction. The primary aim of this study was to measure intravaginal pressure changes using a novel pressure sensor array (Femfit®) during high-risk sports activities in elite female athletes with and without stress urinary incontinence (SUI). The secondary aim was to quantify the amount of urine leakage during these activities using a newly developed innovative pad weighing test (iPWT).

**Methods:**

We used the International Consultation on Incontinence Questionnaire–Urinary Incontinence Short Form (ICIQ-UI SF) to assess participants and measured the VPP using the Femfit® device during ground jumps, trampoline jumps, weightlifting, slow running and fast running. Urine leakage was assessed using the iPWT during the five sports activities.

**Results:**

The highest VPP (53.7 ± 21.6 mmHg) was recorded during ground jumps, followed by trampoline jumps. The second-highest values (24.7 ± 8.1 mmHg) were measured during fast and slow running, while the lowest values were observed during weightlifting (11.6 ± 4.0 mmHg). Measurements of VPP during sporting activities revealed lower pressure values in elite female athletes with SUI than in those without SUI. The overall mean urine leakage in the SUI group during the five activities was 6.6 ± 1.8 g.

**Conclusion:**

On the basis of the VPP and the newly developed iPWT, sports involving jumps and lunges have been identified as revealing SUI and should therefore be included in testing female athletes. The iPWT may prove suitable for measuring SUI in elite female athletes but requires further testing and validation. ClinicalTrials.gov Registration: NCT06224335.

## Introduction

Urinary incontinence is a condition characterised by the involuntary leakage of urine, and the most common type is stress urinary incontinence (SUI). SUI is defined as ‘involuntary loss of urine on effort or physical exertion (e.g. sporting activities), or on sneezing or coughing’ [1].

Sportswomen who participate in high-intensity physical activity experience various health problems during sports activity, including urine leakage [2]. Several studies have confirmed that high-intensity physical activity is a risk factor for SUI [3, 4]. SUI can also occur in younger athletes. We based our selection of high-risk sports activities on a systematic review. High-risk sports activities include trampoline jumping, weightlifting and running [5–9]. Therefore, we selected these high-risk sports activities as provocation manoeuvres in a newly developed pad test.

Normal pelvic floor muscle (PFM) function is defined as a level of constant resting tone, with the ability to voluntarily and involuntarily contract and relax the PFMs. Objective examination of the PFMs is essential. Manometry has been the most commonly used assessment tool in research and clinical settings. Its disadvantage is that it is often tied to the lithotomy position and only measures the resultant pressure from the vaginal canal area. It cannot distinguish between intravaginal and intra-abdominal pressure. Pelvic floor ultrasound imaging measures PFM morphology and function via trans-abdominal, trans-perineal, trans-vaginal and trans-anal placement of the transducer in the lithotomy position [10–13]. For our research, we needed a device that could examine the activity of the PFMs in motion.

The Femfit® device is designed to measure changes in the vaginal pressure profile (VPP) during pelvic floor exercises. The pressure sensor array consists of eight pressure sensors along the length of the device, enabling a VPP to be created in response to changing pressures within the vaginal canal. Pressures are transmitted wirelessly via Bluetooth to a mobile phone, where data can be visualised in real time and uploaded to a secure server. Femfit® allows more targeted measurement of PFM contraction, which is defined as asymmetric, with the zone of strongest contraction occurring 3 to 4 cm from the vaginal opening, mainly in the anteroposterior direction [14–19]. These data are typically obtained by pressure sensors 2 to 4, but this is dependent on posture and anatomical variations. Significant reliability and validity were found when comparing measurements of PFM contraction using Femfit® with a dynamometer, palpation and ultrasound [17].

Measuring changes in the VPP during various sports will help us understand the magnitude of pressures developed and the response of the pelvic floor to those pressures. In turn, this could aid in selecting safe exercises for women with pelvic floor dysfunction. Few studies have been published on measuring VPPs during sporting activities [18, 19]. No study to date has measured urine leakage in grams during high-risk sports activities. A standard pad test does not measure urine leakage at the high intensity of physical activity [20, 21]. Therefore, we decided to develop a new test to quantify urine leakage during several types of high-intensity physical activity in female athletes.

The primary aim of this pilot study was to measure changes in the VPP using Femfit® during high-risk sports activities in elite female athletes with and without SUI and to determine if there were differences between the two groups. The secondary aim was to quantify the amount of urine leakage during the activities using a newly created innovative pad weighing test (iPWT).

## Methods

This observational pilot case-controlled study involved ten elite female athletes, five with and five without SUI, from local sports clubs. For more than 2 years, they had performed sports activities at least 3 days per week for 90 min per day. All athletes carried out sports activities at a competitive level. The presence of SUI was determined using the International Consultation on Incontinence Questionnaire–Urinary Incontinence Short Form (ICIQ-UI SF). Institutional ethics approval was obtained for the study. Each participant had her own Femfit®, which is a single-user device that is self-inserted similarly to a tampon according to the instructions.

### Inclusion Criteria

The following inclusion criteria were used for this study: women who (1) were nulliparous; (2) aged 18–35; (3) engaged in high-intensity physical activity; and (4) performed sport at least 3 days per week, 90 min per day, for more than 2 years.

### Exclusion Criteria

The following exclusion criteria were used for this study: women who (1) were disabled; (2) performed various kinds of sport; (3) had no regular sports activity; (4) performed sports for less than 2 years; (5) had just given birth; (6) had surgical treatment of gynaecological and urological illnesses; (7) presented with urinary tract infection; (8) had a respiratory tract disease; (9) did not sufficiently complete the questionnaires; (10) refused to participate; (11) had a body mass index greater than 30 (BMI = kg/m^2^, where kg = body weight in kilograms and m = body height in metres); and (12) had symptoms of overactive bladder (OAB).

Women with overactive bladder were excluded. None of the participatns had a history of urgency, frequency, nocturia, or urge incontinence. Additionally, the exclusion criteria raise concerns, particularly regarding the exclusion of participants with overactive bladder, given that the questionnaire used (ICIQ-UI SF) is not designed to diagnose this condition.

### Outcome Measures

The primary aim of this study was to measure the VPP using Femfit® during selected sports activities (e.g. jumps on the ground, jumps on a trampoline, weightlifting, slow running and fast running) in elite female athletes with and without SUI and determine if there were differences between the two groups. The secondary aim was to quantify the amount of urine leakage during the activities using a newly developed iPWT.

### Description of Measurement Devices

**Fig. 1:**
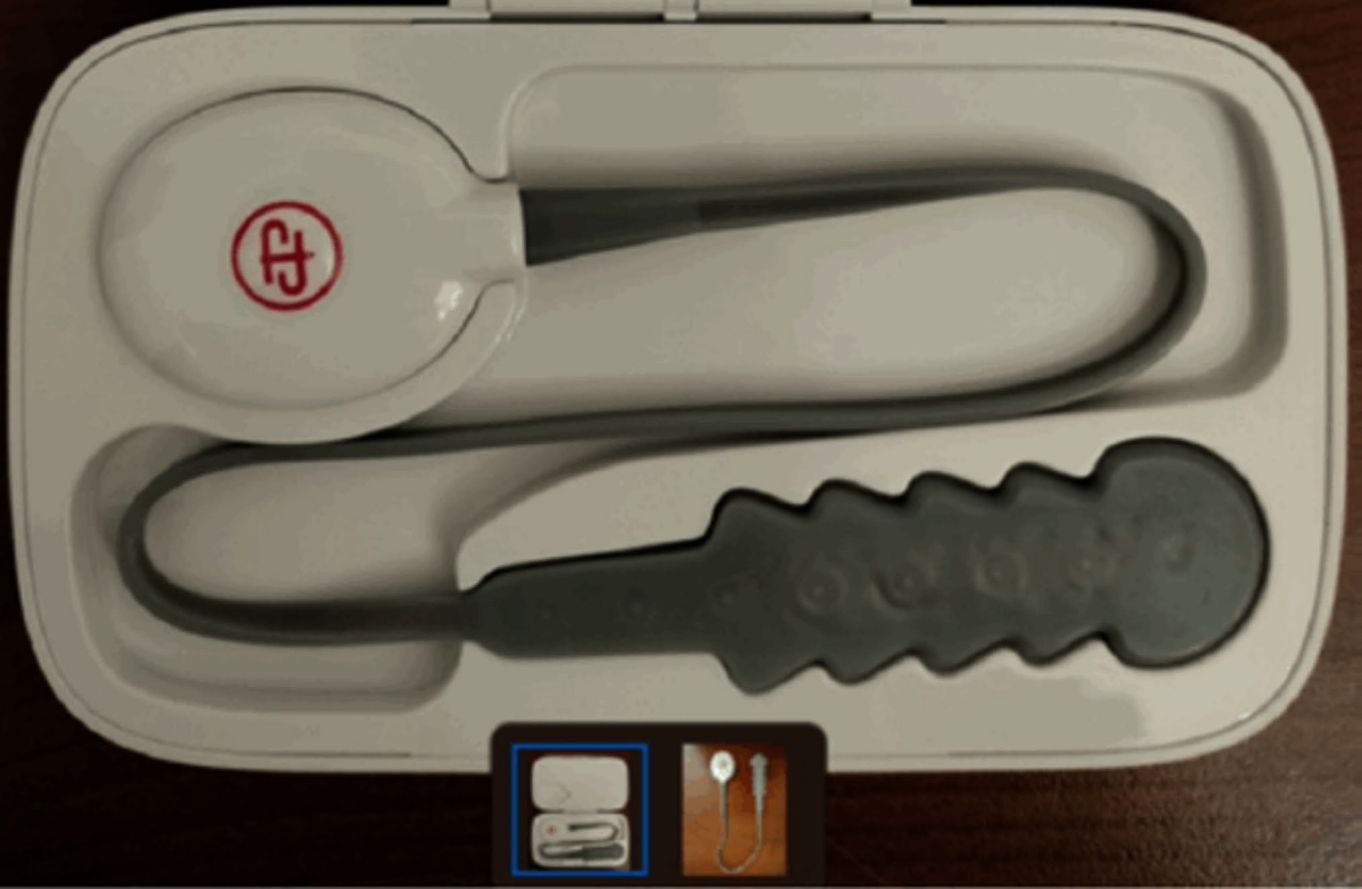
Femfit®

Femfit® (version 2.3.1) is a pressure sensor array designed to measure the pressure profile along the vagina (Fig. 1). It contains eight pressure sensors encapsulated in soft biocompatible silicone (MED-4901; NuSil). Femfit® has a total length of 80 mm, maximum width of 24 mm, and thickness of only 4 mm. These dimensions and the soft flexibility of the device enable pressure measurement without stretching or deforming the vaginal canal. The edges of the device cover a distance of 55 mm and are designed in a way that the device fits snuggly in the vagina, reducing movement of the device in different postures. Pressure data from the Femfit® device is transmitted via Bluetooth to a mobile phone for for real-time display and feedback from the user. Each pressure sensor samples at a frequency of 40 Hz [18, 19, 22].

Significant reliability and validity were found when comparing measurements of PFM contraction using Femfit® with a dynamometer, palpation, and ultrasound (Pearson’s coefficient = 0.72, *p* = 0.006 and Spearman’s rho = 0.68, *p* = 0.005, respectively) [17]. Femfit® is validated to measure VPPs during any physical activity and in motion. During testing, the device was secured with fitted underwear, ensuring its stable position.

### Data Collection

#### Examination of Baseline Pelvic Floor Status with Femfit® at Rest

We used the Femfit® device to examine the baseline status of the PFMs because the device correlates well with dynamometer, palpation, and ultrasound examinations. Femfit® measurements of voluntary pelvic floor contractions were conducted for each participant using one of the programmes available on the Femfit® app. Testing was done in the standing position, with each type of contraction examined for 1 min. Participants performed maximum voluntary contractions, the Knack manoeuvre, rapid contractions and endurance contractions.

Squeeze measurements represent the maximum voluntary contraction that occurs, while knack contractions represent the maximum voluntary contraction under stress (e.g. when someone coughs). Moreover, rapid contractions represent rapid measurements, while endurance contractions provide insight into endurance measurements.

#### Examination of VPP with Femfit® During Physical Activity

Measurements of VPP were carried out during the following sports activities, which were selected as provocative activities in previous studies [9]: jumps on the ground up to 10 cm during 30 s, followed by relaxation for 30 s; jumps on a trampoline up to 20 cm during 30 s, followed by relaxation for 30 s; weightlifting with 25% of body weight (e.g. 60 kg weight of the athlete corresponds to a load of 15 kg) for 30 s, followed by relaxation for 30 s; slow running for 30 s then relaxing for 30 s; and finally, fast running for 30 s and then relaxing for 30 s.

A newly developed iPWT for female athletes with SUI was used to detect urine leakage in grams during the same sporting activities. This test was created by us and used for the first time. The newly developed iPWT was applied to all athletes. It involved emptying the bladder, consuming 500 mL of fluid over 15 min, waiting an additional 15 min, and then performing the sports activities described above. Pads were changed and weighed after each activity. The proposed categories for urine leakage were as follows.

- No SUI if the pad weighed 0 g
- Mild SUI if the pad weighed 1–10 g
- Moderate SUI if the pad weighed 10–50 g
- Severe SUI if the pad weighed > 50 g

Urine leakage was analysed by the increase in pad weight (in grams).

#### The International Consultation on Incontinence Questionnaire (ICIQ-UI SF)

The ICIQ-UI SF was developed by the ICS. The first two questions monitor the frequency and quantity of leaked urine, and the third question considers how urine loss affects daily life. The ICIQ-UI SF score is the sum of the scores from all questions (e.g. 0 = no leak; 1–5 = slight; 6–12 = moderate; 13–18 = severe; and 19–21 = very serious). The Cronbach’s alpha reliability for this questionnaire is 0.95 [23].

### Statistical Analysis

Femfit® data are obtained using eight sensors, each sampling at 40 Hz, resulting in 40 measurements per second. We analysed the difference between the maximum and minimum pressures obtained during a 1-s interval for each sports activity. The average pressure from a 30-s interval for each task, measured by sensors 1 to 8, was used for analysis. Pressure was recorded in units of mmHg. Data are presented as mean and standard deviation, and *p* values were calculated using analysis of variance with Bonferroni corrections and *t*-tests. The significance level was set at *p* < 0.05.

## Results

According to the inclusion and exclusion criteria, the study included ten elite female athletes with an average age of 20 years, recruited from local sports clubs. All participants had a normal weight. For more than 2 years, they had performed sports activities at least 3 days per week for 90 min per day. All athletes engaged in sports activities at a competitive level, including high-risk activities such as jumping and running. Five participants had SUI and five did not, as confirmed by the total score of the ICIQ-UI SF. The groups were homogeneous.

The research participants were also questioned about the movement of the Femfit® during testing. They did not experience movement of the device during testing. The demographic data are shown in Table 1.

**Table 1:** Participant demographics

	Group	Mean	SD	*p*
Age (years)	0	20.20	2.68	0.88
	1	20.00	1.22	
Weight (kg)	0	66.40	11.26	0.43
	1	60.40	11.84	
Height (cm)	0	165.20	8.75	0.35
	1	169.0	3.64	
BMI (kg/m^2^)	0	24.53	5.27	0.27
	1	21.03	4.01	
Years of sport	0	10.0	4.43	0.74
	1	9.40	2.88	
Days of sport	0	4.20	1.09	0.64
	1	4.60	1.51	
Hours of sport	0	2.66	1.44	0.42
	1	2.06	0.70	
ICIQ UI SF- total score	0	0.00	0.00	0.005
	1	6.60	3.13	

0 no urine leakage, 1 with urine leakage

All female athletes were screened for parameters through the Femfit® Starter programme, including squeeze, knack manoeuvre, and fast and endurance contractions during standing.

There were no significant differences between groups with squeeze and fast contractions. For knack contractions, there was a significant difference with sensors 2 and 3 measuring higher pressures in women without SUI. Further, a significant difference was observed for endurance contractions, with sensor 3 measuring higher in women without SUI (Table 2).

**Table 2:** Mean IVP profile (mmHg) during maximal voluntary contraction (squeeze), knack manoeuvre, rapid and endurance contractions

Contraction types		Squeeze	Knack	Rapid	Endurance
Sensors	Group	Mean (SD)	Mean (SD)	Mean (SD)	Mean (SD)
1	0	3.92 (2.16)	4.57 (3.59)	3.75 (3.08)	3.39 (2.66)
	1	2.19 (2.16)	1.64 (0.94)	1.50 (1.61)	1.55 (1.30)
2	0	5.54 (3.15)	5.36 (1.76)*	5.74 (3.97)	5.38 (5.3)
	1	2.60 (2.21)	2.06 (0.88)*	2.30 (2.06)	1.82 (1.25)
3	0	6.75 (1.38)	5.71 (1.44)*	6.30 (1.70)	6.26 (1.73)*
	1	6.36 (2.87)	3.59 (1.15)*	3.97 (1.87)	3.39 (1.52)*
4	0	6.75 (4.55)	5.56 (1.47)	6.52 (2.78)	4.54 (1.38)
	1	6.36 (1.38)	3.74 (1.12)	5.83 (1.28)	4.41 (1.27)
5	0	9.57 (5.23)	6.00 (2.45)	8.82 (6.08)	6.34 (5.12)
	1	7.98 (1.80)	4.98 (1.22)	6.78 (1.34)	4.98 (1.48)
6	0	7.60 (4.87)	6.35 (2.82)	9.00 (8.96)	7.73 (7.28)
	1	7.80 (1.92)	5.35 (1.69)	7.38 (1.54)	4.90 (1.27)
7	0	6.46 (5.01)	6.52 (2.98)	8.00 (8.53)	7.38 (6.82)
	1	7.10 (1.49)	5.41 (1.53)	6.65 (0.93)	5.00 (1.30)
8	0	6.61 (5.23)	6.43 (3.19)	8.16 (10.12)	8.68 (9.44)
	1	7.36 (2.74)	5.60 (2.27)	6.74 (1.40)	4.95 (1.82)

0 no urine leakage, 1 with urine leakage *p < 0.05.

*p*-Analysis of variance with Bonferroni corrections. Nonsignificant results were obtained

Data are from the Starter programme – Examination of current pelvic floor status with Femfit® at rest

Urine leakage was measured in grams with an iPWT test during sports activities. The mean values are shown in Table 3. The ICIQ UI SF questionnaire measured urinary leakage symptoms, and the mean score confirmed moderate urinary leakage.

**Table 3:** Mean urinary leakage in grams during sports activity – in groups of sportswomen with and without SUI

Sports activity	Group	Mean	SD	*p*
Jumps on trampoline	0	0.00	0.00	0.005
	1	1.40	0.54	
Weightlifting	0	0.00	0.00	0.014
	1	1.40	0.54	
Jumps on ground	0	0.00	0.00	0.001
	1	1.20	0.44	
Slow running	0	0.00	0.00	0.005
	1	1.40	0.54	
Fast running	0	0.00	0.00	0.004
	1	1.20	0.44	
Pad test total	0	0.00	0.00	0.007
	1	6.60	1.81	

0 no urine leakage, 1 with urine leakage

The overall urine leakage in the group with SUI from the five activities was 6.6 ± 1.8 g. A mean urine leakage of 1.4 ± 0.5 g was observed during trampoline jumping, weightlifting and slow running. Moreover, a mean urine leakage of 1.2 ± 0.4 g was measured during ground jumping and fast running. No urinary leakage was observed in the group without SUI during the activities.

**Fig. 2:**
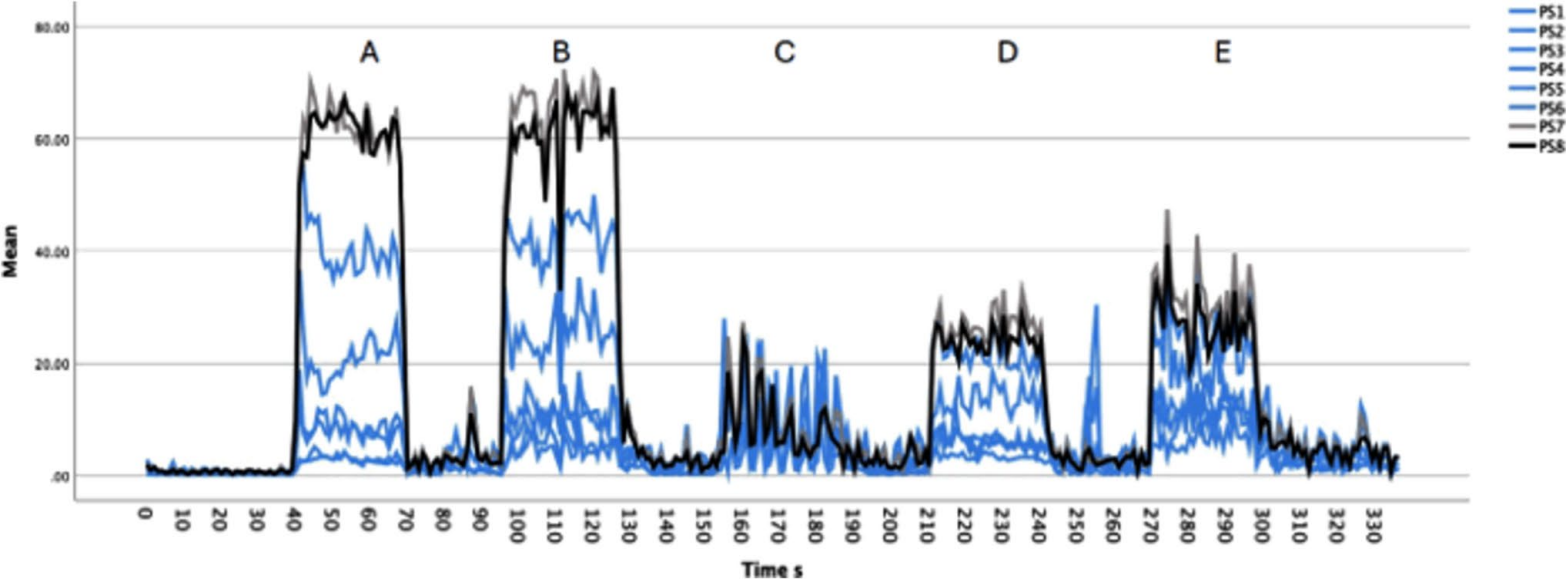
Averaged pressures over the activity for each sensor for all participants. **A** jumps on the ground, **B** jumps on the trampoline, **C** weightlifting, **D** slow running, **E** fast running

Five measurements were obtained from each participant for a total of 50 measurements (Fig. 2).

IVP was recorded with sensors 1–6 and IAP with sensors 7–8 in mmHg. The Femfit® recorded at 40 Hz, and an average was calculated based on the difference between the minimum and maximum pressure per second (Fig. 1).

The average pressure obtained from an interval of 30 s for each task with sensors 1–8 was used for analysis. The highest mean IVP among the ten participants were recorded when jumping on the ground and the trampoline (53.7 ± 21.6 mmHg), followed by fast and slow running (24.7 ± 8.0 mmHg), with the lowest values during weightlifting (11.6 ± 4.0 mmHg). IAP measurements followed the same pattern: the highest observed for jumping on the ground and the trampoline (59.6 ± 14.3 mmHg), followed by fast and slow running (27.1 ± 6.8 mmHg), with the lowest values during weightlifting (10.3 ± 2.5 mmHg). During all sports activities tested, VPP was higher in the group without SUI than in the group with SUI, but the differences were not statistically significant (Tables 4 and 5).

**Table 4:** Average VPP (mmHg) during sports activities, developed by each sensor for each exercise for the whole cohort

Sensor	Jumps on ground	Jumps on trampoline	Weightlifting	Slow running	Fast running
	Mean (SD)	Mean (SD)	Mean (SD)	Mean (SD)	Mean (SD)
1	10.0 (7.9)	6.3 (5.6)	6.8 (8.8)	8.2 (6.3)	18.2 (13.1)
2	14.7 (12.2)	8.2 (6.9)	7.3 (8.5)	9.1 (6.3)	16.5 (9.7)
3	16.6 (11.4)	13.2 (10.2)	5.7 (3.3)	9.7 (7.1)	15.8 (9.2)
4	24.5 (17.8)	15.9 (11.0)	6.6 (2.9)	14.6 (11.6)	17.7 (10.7)
5	37.0 (20.0)	30.8 (16.7)	9.3 (2.6)	16.2 (8.5)	20.1 (9.3)
6	53.7 (21.6)	47.0 (20.2)	11.6 (4.0)	23.1 (7.9)	24.7 (8.0)
7	58.7 (18.9)	52.6 (15.0)	10.5 (2.5)	24.0 (7.0)	27.1 (9.7)
8	59.6 (14.3)	54.4 (11.7)	10.3 (2.5)	25.1 (4.9)	27.1 (6.8)

*p*—Analysis of variance. Nonsignificant differences were obtained

**Table 5:** Average VPP (mmHg) during sports activities, developed by each sensor for each exercise between sportswomen with and without SUI

	Jumps on ground 0	Jumps on ground 1	Jumps on Trampoline 0	Jumps on Trampoline 1	Weightlifting 0	Weightlifting 1	Slow running 0	Slow running 1	Fast running 0	Fast running 1
	Mean (SD)	Mean (SD)	Mean (SD)	Mean (SD)	Mean (SD)	Mean (SD)	Mean (SD)	Mean (SD)	Mean (SD)	Mean (SD)
1	14.4 (9.0)	6.5 (5.6)	7.2 (7.0)	5.4 (4.5)	8.7 (12.5)	4.8 (3.1)	10.1 (8.8)	6.4 (2.3)	23.3 (17.6)	13.1 (3.4)
2	14.5 (12.3)	14.8 (13.6)	9.2 (6.3)	7.3 (8.0)	9.3 (11.9)	5.3 (3.7)	11.1 (7.7)	7.2 (4.4)	20.8 (11.5)	12.2 (5.9)
3	17.9 (11.1)	15.2 (12.7)	13.8 (9.3)	12.7 (12.0)	6.1 (4.0)	5.3 (2.7)	11.4 (8.0)	8.1 (6.5)	18.7 (11.8)	12.9 (5.6)
4	26.9 (21.1)	22.1 (15.8)	14.2 (9.3)	17.6 (13.4)	7.2 (3.7)	6.1 (2.1)	17.0 (14.2)	12.1 (9.3)	22.4 (13.6)	12.9 (4.1)
5	43.0 (18.3)	31.1 (21.8)	34.8 (17.0)	26.8 (17.2)	10.3 (2.5)	8.4 (2.6)	16.9 (10.3)	15.8 (7.3)	22.7 (11.6)	17.5 (6.5)
6	63.6 (22.5)	43.9 (17.3)	56.06 (22.4)	38.0 (14.7)	13.5 (3.4)	9.8 (3.9)	27.3 (8.6)	18.9 (4.8)	27.9 (8.3)	21.6 (7.3)
7	66.7 (21.9)	50.8 (12.8)	58.7 (15.7)	46.4 (12.8)	12.2 (1.5)	8.8 (2.2)	27.9 (7.6)	20.1 (4.0)	31.2 (11.0)	23.1 (7.0)
8	64.8 (17.3)	54.4 (9.8)	59.2 (12.4)	49.7 (9.9)	12.0 (2.1)	8.7 (1.9)	27.1 (4.5)	23.0 (4.8)	30.4 (7.6)	23.9 (4.5)

p—Analysis of variance. Nonsignificant differences were obtained.

0 no urine leakage, 1 with urine leakage. Data are from the Freestyle programme

The highest intravaginal pressures were found for trampolining, measured from sensors (6–8) indicating high IAP – that is, activity when the pelvic floor muscles are most stressed.

## Discussion

High-risk sports activities in our study included trampoline jumping, jumping, weightlifting, and fast and slow running. For our research, we required a device that could examine pelvic floor demand expressed by the VPP during these sports activities.

No study to date has measured urine leakage in grams during standardised high-risk sports activities. We also decided to develop a new pad test to quantify urine leakage during these activities in female athletes. Our female athletes experienced urine leakage only during sports, not during normal activities. No urine leakage would have been observed using the standard pad test. The newly developed iPWT can be used to investigate the association of urine leakage with the VPP.

In the main measurements of our study, we found that as intra-abdominal pressure rose, intravaginal pressure also rose, increasing the load on the pelvic floor. The pressure threshold for developing or worsening pelvic floor dysfunction remains unknown and is likely to vary between individuals.

Measuring the changes in the vaginal pressure profile (VPP) during a variety of sports will help with our understanding of the magnitude of pressures developed during such activities and the response of the pelvic floor to those pressures. In turn, this could aid in selecting safe exercises for women with pelvic floor dysfunction. Few studies have been published on measuring VPPs during sporting activities [15–19]. No study to date has measured urine leakage in grams during high-risk sports activities using a newly created pad test. Only standard pad tests have been used, but they do not assess high-intensity physical activity [20, 21]. High-risk sports activities in our study included trampoline jumping, jumping, weightlifting, and fast and slow running. We selected these activities based on a systematic review and meta-analysis [9].

The primary aim of this pilot study was to measure changes in the VPP using the Femfit® during high-risk sports activities in elite female athletes with and without SUI and to determine if there were differences between the two groups. The secondary aim was to quantify the amount of urine leakage during the activities using the newly created iPWT.

In our cohort, we had ten nulliparous female athletes with a mean age of 20 years who engaged in high-intensity physical activity.

The results suggest that the ability of the pelvic floor to respond sufficiently to increases in IAP during high-intensity activity appears to be reduced in athletes with SUI. The higher intravaginal pressures measured while jumping on the trampoline corresponded to the most urine leakage in athletes with SUI, which is unsurprising because previous research has shown that female trampolinists often experience the highest incidence of SUI. Jumping on a trampoline causes a very strong continuous increase in intra-abdominal pressure [17].

Interestingly, lower IVP overall was observed for weightlifting (25% of body weight), yet those athletes with SUI still had a higher volume of leakage during this activity, suggesting there is perhaps a threshold for leakage that is dependent on more than just pressure generation. However, these results should be interpreted with caution due to the small sample size.

In an observational prospective study, Kruger [18] measured vaginal pressures with Femfit® in 24 women with a mean age of 37.5 years during exercises that may also have a negative impact on the pelvic floor. This study evaluated planks, bicycling, running, walking, push-ups, lunges and squats. Running, push-ups, squats and standing cycling produced higher pressures on all sensors than equivalent safe pelvic floor exercises. IVP was highest during running. However, the pressure threshold for the development or worsening of pelvic floor dysfunction remains unknown and is likely to be individual-specific. In our study, we observed the highest IVP with ground and trampoline jumps.

Cacciari [17] tested intravaginal pressure changes using Femfit® in 20 healthy women during the contraction of the PFMs and during the Valsalva manoeuvre in the supine and standing positions. They were compared with maximal voluntary contraction assessed by dynamometry and palpation through the vagina. A positive significant correlation was found between IVP and both dynamometry and vaginal palpation.

Shaw [24] measured IAP in 57 women while performing different types of physical activity using a different measuring device – The Gen2 Intravaginal transducer. The women performed 31 sports activities at light, moderate and high intensities. IAP was measured during coughing, Valsalva, lying down, sitting and during aerobic activities, including walking, running, cycling and stepping. Other measurements were taken while performing daily activities, lifting tasks, stabilisation exercises and stretching activities. An important finding was that coughing had a high maximum pressure of 199.9 cmH_2_O (153.7 mmHg) than the other activities. The seated Valsalva had a similarly high maximum pressure of 207.7 cmH_2_O (159.7 mmHg) compared to the other activities. In our study, we recorded the highest average 64.8 mmHg, with ground and trampoline jumps.

These results imply that it is important to recognise the magnitude of IAP but also the response of the pelvic floor to that increase in pressure.

The secondary aim of this study was to quantify the amount of urine leakage using an iPWT.

The meta-analysis showed a 25.9% prevalence of urinary incontinence in female athletes in different sports, as well as a 20.7% prevalence of SUI. The most prevalent high-impact sport was volleyball, with a value of 75.6%, then jumps on the trampoline at 72.7% and running at 44.0%. On the basis of the above meta-analysis, we selected the most risky movement activities for the iPWT [9].

Several authors have used a standard 1-h pad test in their studies, including slow walking, standing from sitting, coughing, running in place, standing from lying down and hand washing. The pad is weighed after the entire test, not after each activity. Except for coughing, it did not include any activity that would significantly increase IAP [25].

We measured urinary leakage symptoms with the ICIQ UI SF questionnaire, and the mean score confirmed moderate urinary leakage. The overall sum of the mean urine leakage measurements from the five sports activities was 6.60 g. We created an iPWT to test urine leakage during each sports activity mentioned above. Five measurements were taken for each patient; thus, the average values were obtained from 50 measurements. On average, 1.40 g of urine leakage was measured during each activity: trampoline jumping, weightlifting and slow running. Meanwhile, 1.20 g urine leakage, on average, was measured during ground jumping and fast running.

### Limitation and Strengths

The primary limitation of this study is the small number of participants, which means the conclusions should be interpreted with caution. The study’s strengths include the development of the newly created iPWT and its application during five types of high-risk sports activities. Another strength is the use of a device capable of examining pelvic floor demand, as expressed by the VPP, during these sports activities in female athletes with and without SUI.

## Conclusion

A unique capability of the Femfit® device is its ability to measure intravaginal pressure along the entire length of the vagina during any physical activity. Measurements of VPP during sporting activities revealed lower pressure values in elite female athletes with SUI than in those without SUI. On the basis of the VPP and the newly developed iPWT, sports involving jumps and lunges have been identified as revealing SUI and should therefore be included in testing female athletes. The newly developed iPWT may prove suitable for measuring SUI in elite female athletes, but further testing and validation are required.

## References

[1] Haylen BT, de Ridder D, Freeman RM, et al. An International Urogynecological Association (IUGA)/International Continence Society (ICS) joint report on the terminology for female pelvic floor dysfunction. Int Urogynecol J. 2010;21:5–26. 19937315 10.1007/s00192-009-0976-9

[2] Jean-Baptiste J, Hermieu JF. Sport and urinary incontinence in women. Prog Urol. 2010;20:483–90. 20656269 10.1016/j.purol.2010.02.007

[3] Almeida MB, Barra AA, Saltiel F, et al. Urinary incontinence and other pelvic floor dysfunctions in female athletes in Brazil: a cross-sectional study. Scand J Med Sci Sports. 2016;26:1109–16. 26369504 10.1111/sms.12546

[4] Simeone C, Moroni A, Pettenò A, et al. Occurrence rates and predictors of lower urinary tract symptoms and incontinence in female athletes. Urologia. 2010;77:139–46. 20890872

[5] Fozzatti C, Riccetto C, Herrmann V, et al. Prevalence study of stress urinary incontinence in women who perform high-impact exercises. Int Urogynecol J. 2012;23:1687–91. 22618204 10.1007/s00192-012-1786-z

[6] Bø K, Sundgot-Borgen J. Are former female elite athletes more likely to experience urinary incontinence later in life than nonathletes? Scand J Med Sci Sports. 2010;20(100–104):18. 10.1111/j.1600-0838.2008.00871.x 19000097

[7] Dias N, Peng Y, Khavari R, et al. Pelvic floor dynamics during highimpact athletic activities: a computational modeling study. Clin Biomech. 2017;41(20–27):19. 10.1016/j.clinbiomech.2016.11.003 PMC5519824 27886590

[8] Nygaard I, Shaw J, Egger MJ. Exploring the association between lifetime physical activity and pelvic floor disorders: study and design challenges. Contemp Clin Trials. 2012;33:819–27. 22521947 10.1016/j.cct.2012.04.001 PMC3361559

[9] Pires T, Pires P, Moreira H, Viana R. Prevalence of urinary incontinence in high-impact sport athletes: a systematic review and meta-analysis. J Hum Kinet. 2020;21(73):279–88. 10.2478/hukin-2020-0008 PMC7386138 32774559

[10] Frawley H, Shelly B, Morin M, Bernard S, Bø K, Digesu GA, Dickinson T, Goonewardene S, McClurg D, Rahnama’i MS, Schizas A, Slieker-Ten Hove M, Takahashi S, Voelkl Guevara J. An International Continence Society (ICS) report on the terminology for pelvic floor muscle assessment. Neurourol Urodyn. 2021;40(5):1217–60. 33844342 10.1002/nau.24658

[11] Rogers RG, Pauls RN, Thakar R, et al. An International Urogynecological Association (IUGA)/International Continence Society (ICS) joint report on the terminology for the assessment of sexual health of women with pelvic floor dysfunction. Neurourol Urodyn. 2018;37(4):1220–40. 29441607 10.1002/nau.23508

[12] Raizada V, Bhargava V, Jung S-A, et al. Dynamic assessment of the vaginal high-pressure zone using high-definition manometery, 3-dimensional ultrasound, and magnetic resonance imaging of the pelvic floor muscles. Am J Obstet Gynecol. 2010;203(2):172.e1-172.e8. 20462564 10.1016/j.ajog.2010.02.028 PMC2910785

[13] Cacciari LP, Pássaro AC, Amorim AC, et al. Novel instrumented probe for measuring 3D pressure distribution along the vaginal canal. J Biomech. 2017;58:139–46. 28549600 10.1016/j.jbiomech.2017.04.035

[14] Rosenbluth EM, Johnson PJ, Hitchcock RW, et al. Development and testing of a vaginal pressure sensor to measure intra-abdominal pressure in women. Neurourol Urodyn. 2010;29(4):532–5. 19693948 10.1002/nau.20794 PMC3731207

[15] Hamad N, Shaw J, Nygaard I, et al. More complicated than it looks: the vagaries of calculating intra-abdominal pressure. J Strength Cond Res. 2013;27(11):3204–15. 23439349 10.1519/JSC.0b013e31828b8e4c PMC3710314

[16] Hsu Y, Coleman TJ, Hitchcock RW, et al. Clinical evaluation of a wireless intra-vaginal pressure transducer. Int Urogynecol J Pelvic Floor Dysfunct 2012;23(12):1741-7. 10.1007/s00192-012-1811-2 PMC3725759 22618208

[17] Cacciari LP, Kruger J, Goodman J, et al. Reliability and validity of intravaginal pressure measurements with a new intravaginal pressure device: the FemFit®. Neurourol Urodyn. 2020;39:253–60. 31588623 10.1002/nau.24179

[18] Kruger J, Budgett D, Goodman J, et al. Can you train the pelvic floor muscles by contracting other related muscles? Neurourol Urodyn. 2019;38(2):677–83. 30592502 10.1002/nau.23890

[19] Kruger J, Budgett D, Goodman J, et al. Measuring the vaginal pressure profile during exercise. Aust N Z Cont J. 2018;24(3): p85.

[20] Karantanis E, O’Sullivan R, Moore KH. The 24-hour pad test in continent women and men: normal values and cyclical alterations. BJOG. 2003;110(6):567–71. 12798473

[21] Figueiredo EM, Gontijo R, Vaz CT, Baracho E, da Fonseca AM, Monteiro MV, Filho AL. The results of a 24-h pad test in Brazilian women. Int Urogynecol J. 2012;23(6):785–9. 10.1007/s00192-011-1645-3 22398823 10.1007/s00192-011-1645-3

[22] McConnell J, Murtagh L, Lim M, et al. A pilot study to evaluate changes in pelvic floor muscle tone following pelvic organ prolapse surgery using a novel intra-vaginal pressure sensor device. Int Urogynecol J. 2023;34(5):1043–7. 35939097 10.1007/s00192-022-05312-4 PMC10167131

[23] Avery K, Donovan J, Peters TJ, et al. ICIQ: a brief and robust measure for evaluating the symptoms and impact of urinary incontinence. Neurourol Urodyn. 2004;23:322–30. 15227649 10.1002/nau.20041

[24] Shaw JM, Hamad NM, Coleman TJ, et al. Intra-abdominal pressures during activity in women using an intra-vaginal pressure transducer. J Sports Sci. 2014;32(12):1176–85. 24575741 10.1080/02640414.2014.889845 PMC3992988

[25] Ferreira S, Ferreira M, Carvalhais A, et al. Reeducation of pelvic floor muscles in volleyball athletes. Rev Assoc Med Bras. 2014;60:428–33.

